# Trylons: Polyamide Surrogate Substrates Enable High‐Throughput Screening of Nylon‐Degrading Enzymes

**DOI:** 10.1002/cbic.70459

**Published:** 2026-07-08

**Authors:** Alana M. M. Rangaswamy, Francis M. Roy, Maria E. Cleveland, Jeffrey W. Keillor

**Affiliations:** ^1^ Department of Chemistry and Biomolecular Sciences University of Ottawa Ottawa Ontario Canada; ^2^ Department of Chemistry Queen’s University Kingston Ontario Canada

**Keywords:** assay development, biocatalysis, nylon hydrolase, polyamide, protein engineering

## Abstract

The aminohexanoate oligomer hydrolase enzymes, NylCs, have emerged as lead biocatalysts for their potential to depolymerize nylon in a bioremediation context. Protein engineering efforts have thus far improved thermostability and activity, but conversion of the polymer to smaller oligomers remains low. With the intent to accelerate engineering efforts, we report the design, synthesis, and application of polyamide surrogate substrates to a rapid, high‐throughput screen of NylC activity by continuous light scattering measurements. Evaluation of a small panel of diamide substrates revealed unprecedented specificity for the amide bonds recognized and hydrolysed by NylCs. We further demonstrate that the active substrates, Trylon‐6 and Trylon‐66, act as surrogates for nylon hydrolysis, where activity of NylCs towards Trylon is predictive of their activity towards bulk nylon film. Finally, we apply the surrogate screen to four mutant libraries of NylC_K_ TS, where activity of the libraries (nearly 100 variants each) was determined in 20 min. From this screen, we report substitution tolerances at positions 146, 189, 192, and 305, identifying residues near the active site of NylC enzymes that are necessary for amidase activity.

## Graphical abstract

The NylCs are nylon hydrolases currently at the forefront of research investigating biocatalysts for nylon degradation. Protein engineering of NylCs has been limited by the inefficiency of activity assays for the evaluation of large mutant libraries. Here, we describe the application of small nylon analogue substrates to a high‐throughput screen allowing for continuous quantification of polyamidase activity by NylCs.

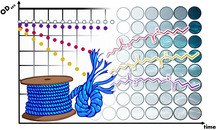

## 1 Introduction

Nylon, a category of plastic defined by its aliphatic, linear polyamide structure, is ubiquitous in industry and commercial applications due to its high tensile strength, flexibility, and resistance to both chemical and physical wear [1]. However, due to this inherent stability, nylon waste is resistant to natural degradation and consequently accumulates in the environment [2]. This accumulation is also a reflection of recycling practices that are not optimized for nylon degradation, where the primary recycling method, thermomechanical processing, leads to an overall decrease in material quality while also requiring significant energy input [3]. An alternative to traditional recycling is depolymerization, which has been done chemically at lab and small industrial scale [4, 6], returning monomers to be re‐polymerized or otherwise valorized into new materials. While this avenue shows promise, it currently requires extreme temperature and pressure conditions and/or expensive metal catalysts, corresponding to a potentially unsustainable energy input. Taken together, these recycling strategies are insufficient to meet current nylon recycling needs and less than 5% of nylon waste is recycled [7], highlighting the need for new technologies to manage this waste.

Enzymatic depolymerization has been proposed as a powerful tool to facilitate efficient and environmentally sustainable plastic recycling under mild conditions, and great strides have been made towards industrial‐scale depolymerization of polyesters such as PET [8, 10]. Similarly, although the stability of the amide bond poses an additional challenge to depolymerization, development of polyamide‐hydrolysing biocatalysts is a rapidly evolving field. Bacteria with aliphatic amide hydrolysis capability were identified as early as the 1960s [11], leading to the characterization of an enzymatic pathway for the degradation of nylon oligomers [12]. Instrumental in this pathway are the NylCs, first identified in *Agromyces* sp. KY5R (NylC_A_) [13], *Kocuria* sp. KI725 (NylC_K_) [13], and *Arthrobacter* sp. KI72 (NylC_p2_) [14]. These enzymes are nylon oligomer endohydrolases, recognizing only oligomers of three or more units [14] and demonstrating some activity against bulk nylon, preferentially releasing dimers of 6‐aminohexanoate following incubation with Nylon‐6 [12, 15]. Since their discovery, the NylC enzymes have served as the basis for both the discovery of novel nylon‐degrading enzymes [16, 17] and engineering efforts to improve catalytic properties [18, 21]. These latter campaigns have thus far led to rationally engineered highly thermostable NylC variants [18, 20], allowing for longer reaction times at elevated temperatures, which are valuable qualities for plastic‐degrading enzymes. Meanwhile, engineering through random and guided mutagenesis has led to improvements in catalytic activity [19, 21] although the extent of depolymerization to lower oligomers remains low (<1%).

Engineering by directed evolution is a powerful strategy for the development of tuned, potent biocatalysts, but is limited by the efficiency of the activity screen employed. A rapid, high‐throughput, continuous, and quantitative assay permits screening of larger libraries with a correspondingly higher probability of identifying an enzyme variant with improved catalytic activity. Unfortunately, since amide hydrolysis does not correspond to a continuously detectable output (such as the generation of a chromophore), current assays require extended reaction times and discontinuous methods to quantify reaction progress. One such class of assays relies on spectrophotometric evaluation of the production of free amines via a secondary reaction between the generated amines and chromogenic electrophiles [12, 19]. This approach overcomes the challenges of quantifying bulk nylon depolymerization, but is discontinuous, requiring long reaction times and extensive manipulation of samples in order to analyse large libraries and complicating the determination of reaction kinetics. Quantification of reaction progress can also be achieved by mass spectrometry techniques [20, 22], which provide more information about reaction products, but are much lower throughput or require specialized automation, representing a bottleneck to protein engineering efforts.

In this context, inspired by substrate analogues used to great effect to identify PETase activity [23, 24], and turbidity‐based assays for plastic‐degrading enzymes [12, 25, 26], we describe the design and application of small‐molecule nylon analogue substrates for nylonase activity screens. These substrates allow for polyamidase activity to be monitored continuously and quantitatively by light scattering, in an assay translatable to high‐throughput screening. We apply this screen to libraries containing hundreds of nylonase variants in minutes to hours, identifying substitution profiles necessary for retention of catalytic activity at substrate‐exposed positions. Finally, varying the structure of the small‐molecule substrates revealed unprecedented substrate specificity in the known wild‐type and engineered thermostable NylC variants, which could provide future directions for engineering to improve substrate tolerance for different nylon compositions.

## 2 Results and Discussion

### 2.1 Evaluation of Amidase Activity Using Nylon Analogues

**FIGURE 1:**
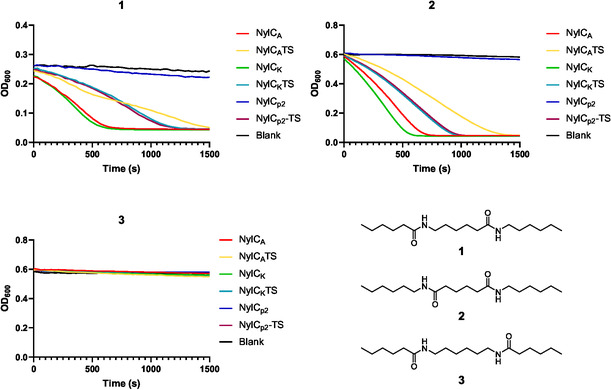
Structure of nylon analogue substrates and hydrolysis by NylC enzymes. Reactions were performed at 50 °C in reaction mixtures containing buffer (20 mM KH_2_PO_4_ pH 7.3, 15% glycerol), BSA (0.1 mg/mL), substrate (1 mM), methanol (5% v/v), and NylC enzyme (0.1 mg/mL). Blank reactions contain no enzyme. Reactions were performed in triplicate, but one replicate from each enzyme is shown for clarity.

Since NylC is an oligomer hydrolase, recognizing substrates of three or more aminohexanoic acid units, we synthesized three analogue substrates, compounds **1**–**3**, each bearing two internal amide bonds (Figure 1). At assay concentration (1 mM), these substrates demonstrate low solubility and form turbid suspensions; however, upon hydrolysis of either amide bond, highly soluble products are formed and the suspension clarifies. In this way, hydrolysis can be easily and continuously monitored by the decrease in light scattering over time, as measured by optical density (OD_600_) as the insoluble substrate is consumed. These substrates were designed to mimic Nylon‐6 (**1**, bearing an aminohexanoic acid unit) or Nylon‐66 (**2** and **3**, bearing adipoyl or hexamethylenediamine units, respectively), the two most industrially relevant nylon polymers. Compound **2** has previously been reported as a nylon analogue substrate [24], but not for the NylC enzymes, and not in the context of a turbidimetric assay as described here.

Compounds **1–3** were evaluated as substrates for each NylC homologue (Figure 1). While we observed a steady decrease in OD_600_ over time as compounds **1** and **2** were consumed by NylC, we noted that compound **3** retained a constant OD_600_ over time, consistent with that of the blank containing no enzyme. This suggested that compound **3** is not recognized by the NylC enzymes—a surprising result given that compounds **1–3** are isomers, differing only in the orientation of their respective amide bonds. We hypothesized that NylC exhibits selectivity for hydrolysis at “*C*‐terminal” amide bonds, where the amine component of the amide bond is oriented at the outside of the peptide chain, since both substrates **1** and **2** bear this feature.

**FIGURE 2:**
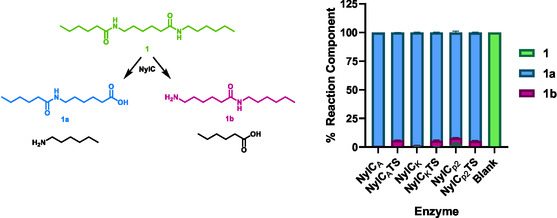
Analysis of compound 1 hydrolysis products by LCMS. Reaction products were analysed following incubation of **1** (1 mM) with NylC (0.1 mg/mL) at 50 °C for 90 min. Error bars represent standard error of two replicates.

To investigate the specificity of NylC towards each amide bond, the hydrolysis products of the asymmetric substrate **1** were evaluated by UPLC‐MS (Figure 2). All enzymes demonstrated high selectivity for *C*‐terminal hydrolysis under both conditions, with the highest selectivity demonstrated by NylC_A_ and NylC_K_ (>99% based on product ratio). Although slightly higher promiscuity was observed by the thermostable enzymes, all homologues demonstrated a selectivity of >94%. This result is consistent with our observation that the *N*‐terminal amide bonds of **3** are apparently not hydrolysed by NylC (see above), revealing a heretofore unknown substrate selectivity for this class of enzyme.

This specificity, which seems to be driven by the orientation of the scissile amide bond, could also shed light on specificity observed during hydrolysis of different nylon polymers by the NylCs. As of now, these enzymes are thought to follow endo‐type hydrolysis, preferentially releasing dimers of 6‐aminohexanoic acid from Nylon‐6 [20, 27] and “monomers” of Nylon‐66 which contain an amide‐linked hexanediamine and adipoyl unit [28]. Based on our small‐molecule substrates, amide bond orientation may have a role to play during nylon hydrolysis, where, for instance, only every other bond is cleaved in the Nylon‐66 polymer if the enzyme acts processively. Furthermore, generation of a charged moiety (carboxylate) in the vicinity of the only scissile bond may hinder binding to the hydrophobic catalytic pocket. This could also explain why we did not observe consumption of compound **1a** (Figure 2).

**FIGURE 3:**
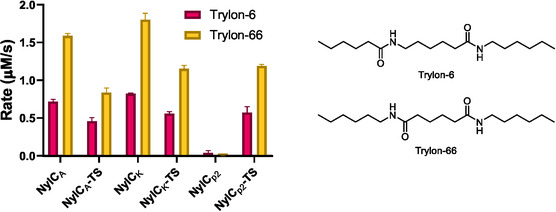
Rates of hydrolysis of Trylon‐6 and Trylon‐66 by NylC homologues. Reactions were performed at 50 °C in reaction mixtures containing buffer (20 mM KH_2_PO_4_ pH 7.3, 15% glycerol), BSA (0.1 mg/mL), substrate (1 mM), methanol (5% v/v), and enzyme (0.1 mg/mL). Reaction rates were determined by converting the negative initial slope of each measurement (OD_600_ over time) to units of concentration over time using the calibration curves shown in Figure S1. Datapoints represent averages of three replicates, with error bars indicating standard deviations.

The rates of NylC‐mediated hydrolysis of compounds **1** and **2**, named *Trylon‐6* and *Trylon‐66* respectively due to their structural analogy to their corresponding polymers, were quantified by correlating the OD_600_ to units of concentration (see Figure S1). Corrected rates for the reaction of each substrate by each NylC enzyme are shown in Figure 3. NylC_A_ and NylC_K_ demonstrated the highest activity towards both substrates, consistent with the observations of Yasuhira et al., who observed faster hydrolysis of aminohexanoate cyclic oligomer by NylC_K_ and NylC_A_ compared to NylC_p2_ [13]. It should also be noted that NylC_p2_ has a melting temperature of 52 °C [18] and is likely inactivated at the reaction temperature of 50 °C, hence the negligible observed activity here. The rates of hydrolysis by the thermostable enzymes are similar to each other and lower than the corresponding mesophilic enzymes (with the exception of NylC_p2_). This is a known phenomenon among thermostable enzymes at low temperatures [29] which are generally less flexible and mobile than their mesophilic counterparts. Therefore, we would expect to see higher activity from NylC_A_ and NylC_K_ than their respective thermostable derivatives at 50 °C, which is sufficiently below their melting temperatures (60 °C and 67 °C, respectively [18]) to retain activity.

All of the enzyme homologues evaluated appear to demonstrate a preference for Trylon‐66 compared to Trylon‐6, at an approximately 2.3‐fold higher rate across all enzymes (see Figure S2). This apparent preference could be due to the affinity of the enzyme for the substrate, or because, as demonstrated above, Trylon‐66 has two scissile bonds compared to only one in Trylon‐6, thus any binding interaction would be productive.

### 2.2 Nylon and Trylon Hydrolysis as Amidase Activity Reporters of NylCs

Hydrolysis of Trylon is fast, convenient, and adaptable to high‐throughput screens of potential nylon‐degrading enzymes. However, to confirm that Trylon hydrolysis relates to nylon degradation, we also evaluated the rates of hydrolysis of Nylon‐6 and Nylon‐66 film by all six NylC enzymes using the previously reported TNBS assay [13]. Standardized nylon films were sourced from Goodfellow, and crystallinity was determined by differential scanning calorimetry (24.7 ± 0.6% for Nylon‐6 and 22.9 ± 0.4% for Nylon‐66). Hydrolysis was evaluated over 24 h, and initial rates were determined from the linear region of the resulting kinetic curves (see Figure S3).

Nylon‐66 is a desirable target for depolymerization as it is not recycled industrially, and the majority of studies investigating enzymatic nylon depolymerization have targeted Nylon‐6. Consequently, while studies investigating novel enzymes have placed an increasing emphasis on enzymatic activity towards Nylon‐66 [16, 17, 21], this comparison has not previously been performed with existing nylonases, save for one report that NylC_p2_TS demonstrated ∼60% activity towards Nylon‐66 powder compared to Nylon‐6 powder [15]. Here, we observe that the rates of Nylon‐6 and Nylon‐66 hydrolysis by all NylCs are relatively similar (see Table S1) where, depending on the enzyme, NylCs demonstrated 66%–91% activity towards Nylon‐66 compared to Nylon‐6. Furthermore, the wild‐type enzyme NylC_K_ demonstrates the highest activity towards the polymers among all of the evaluated enzymes. As previously discussed, the mesophilic enzymes are expected to demonstrate higher activity than their thermostable counterparts up to their denaturation temperatures. This results in little to no activity in NylC_p2_ with a melting temperature of 52 °C [18], but it is interesting to note that NylC_K_ retains the highest activity among all enzymes, even up to 24 h at 50 °C. While we chose 50 °C to match the Trylon hydrolysis conditions, the thermostable enzymes have previously been reported to have higher optimal temperatures of ∼70 °C [20] at which the observed trend would likely change to favour the thermostable enzymes.

**FIGURE 4:**
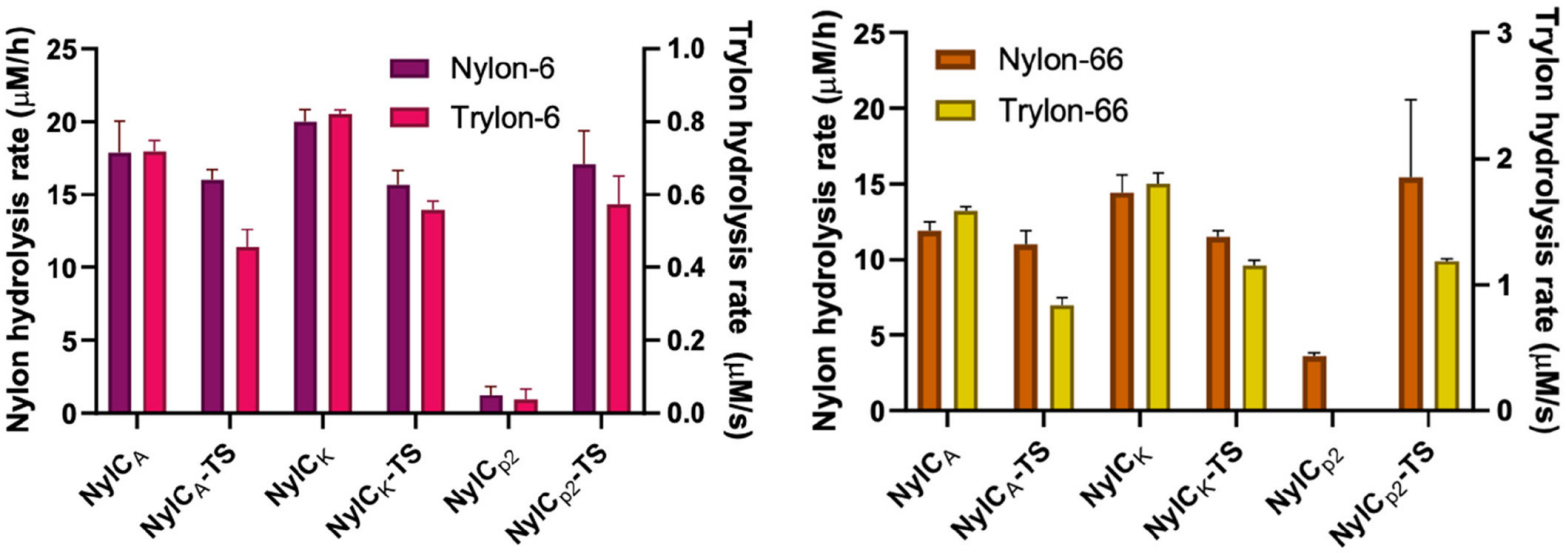
Comparison of the relative rates of hydrolysis nylon film (left *y*‐axes) and their corresponding Trylon substrates (right *y*‐axes) for the polymers, Nylon‐6 (left graph) and Nylon‐66 (right graph). Nylon hydrolysis rates are reported as concentration of free amines produced over time, as quantified by TNBS. Reaction conditions for nylon hydrolysis: NylC (0.1 mg/mL), potassium phosphate buffer (20 mM, pH 7.3), and nylon film (Nylon‐6, 8.2 mg; Nylon‐66, 18.1 mg). Values for nylon hydrolysis are averages of two replicates, with error bars showing standard deviations.

To determine the relationship between Nylon and Trylon hydrolysis, we compared the relative rates of Nylon hydrolysis to those of the structurally analogous Trylon substrates (Figure 4). While the rate of Trylon hydrolysis is ∼150‐fold higher than that of the corresponding nylon hydrolysis, the relative rates between the enzymes are similar for the substrates, with NylC_A_ and NylC_K_ again demonstrating the highest activity against all substrates. We observe a greater discrepancy in relative rates with the thermostable enzymes, where the activity is higher against nylon than would be expected on the basis of Trylon hydrolysis rates. While reactions with both polymer and small‐molecule substrates are conducted at 50 °C, the enzymes need only retain activity against Trylon for approximately 30 min, compared to the corresponding reactions with Nylon, where activity is measured over 24 h. The thermostable enzymes are active for longer at elevated temperatures relative to their non‐thermostable counterparts; thus, the relative activities observed for nylon hydrolysis by the thermostable enzymes are higher than Trylon would predict.

### 2.3 High‐Throughput Screening Using Trylon

**FIGURE 5:**
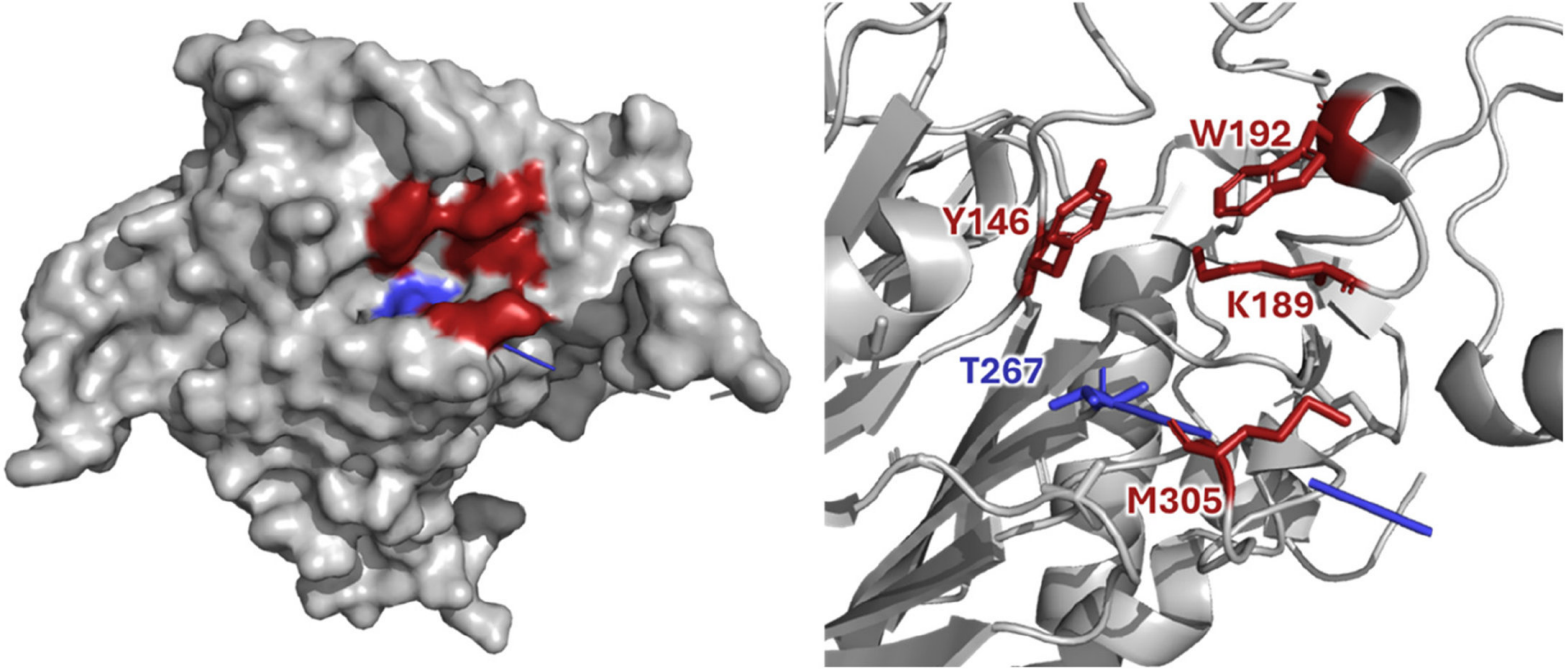
Left: surface of a NylC_A_ heterodimer subunit at the active site. Right: residues selected for saturation mutagenesis are in red; the catalytic threonine residue is in blue. PDB ID: 3AXG, visualized using PyMol.

To demonstrate the use of Trylon as a screen for amidase activity in NylC‐type enzymes, we performed a series of site‐saturation mutagenesis experiments, targeting residues at the surface of the active site surrounding the catalytic threonine. We hypothesized that these residues would be implicated in substrate binding, and thus, hydrolysis activity would be sensitive to mutations at these positions. Furthermore, positions 146 and 189 have previously been investigated for their participation in amide hydrolysis, where alanine mutants at each of those positions led to reduced autoprocessing (6%–10% for K189A, 37% for Y146A) and reduced activity (0.6% for K189A and 3.6% for Y146A) compared to NylC_p2_TS [30]. Thus, we considered that mutations at these positions may lead to zero activity in all but the wild‐type enzyme, which would be reflected in the screen. Figure 5 shows the active site (nylon‐binding) groove of NylC_A_ and the location of Y146, K189, W192, and M305, the conserved residues chosen for this study. We chose NylC_K_TS as the basis for mutagenesis, reasoning that selecting a thermostable variant as the starting point for mutagenesis studies decreases the likelihood that any one mutation would lower the melting temperature below a useful temperature for nylon hydrolysis.

Four libraries (one for each residue) were prepared by full‐plasmid PCR using the degenerate codon, NNK, representing all 20 amino acids [31]. The circularized amplicons were transformed into *E. coli* which was cultured on agar plates. For adequate oversampling, 90 of the resulting colonies were selected (94% coverage). Each colony was used to inoculate one well of a single deep‐well 96‐well plate, leaving six wells for controls: wild‐type enzyme (positive control), empty vector (negative control), and no cells (to verify sterility) in duplicate. Thus, each library was thoroughly and conveniently screened in one plate.

Cell lysate was prepared from the resulting 96 × 1 mL expression cultures and evaluated for activity by clarification of Trylon‐6 at 50 °C over 30 min. This period is longer than the requisite 20 min for full clarification of Trylon‐6 by purified NylC_K_TS, so as to account for potential variable expression levels between different cell lysates. However, wells containing active enzyme were identifiable by an immediate decrease in OD_600_ (Figure S4a–d). Select wells representing both active and inactive mutants were sequenced from each plate to ensure that the observed differences in activity were due to successful mutations, and not simply variable expression levels of the wild‐type enzyme.

Across four libraries, we identified eight mutations that led to observable amidase activity. At position 189, we identified no active mutants, consistent with the findings of Negoro et al. [30]. However, we did observe one active mutant at position 146: while Negoro only evaluated Y146A, our F146 mutant showed similar activity to NylC_K_TS in the screen, suggesting that an aryl residue may be necessary to retain activity. Positions 192 and 305 each demonstrated greater tolerance for mutations, with three and four active mutants identified at each position, respectively. While position 192 favoured bulky, hydrophobic residues (Leu, Phe, Met), position 305 was more variable still, showing activity with residues including His, Leu, Cys, and Val. A concurrent study by Puetz et al. also identified position 305 as being non‐conserved among active NylC variants [21]. Notably, while many different residues are tolerated at this position, most mutations led to zero activity. As an example, despite its similarity to active mutants V305 and C305, the mutant T305 was inactive, indicating that this position is nonetheless sensitive to small structural changes.

**FIGURE 6:**
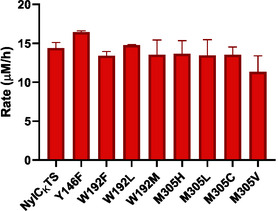
Hydrolysis of Nylon‐6 film by NylC_K_TS single mutants. Nylon hydrolysis rates are reported as concentration of free amines produced over time, as quantified by TNBS. Reactions were performed at 50 °C for 24 h and contained NylC (0.1 mg/mL), potassium phosphate buffer (20 mM, pH 7.3), and Nylon‐6 film (8.2 mg). Values are averages of three replicates, with error bars showing standard deviations.

The active mutants of NylC_K_TS identified through the Trylon screen were purified and evaluated for Nylon hydrolysis activity (Figure 6). We were pleased to observe notable nylonase activity in all of the active mutants identified in the screen. The reaction rates from crude lysate were more variable among mutants than those of the reactions with purified enzyme, which were all similar to the wild type. This could be attributed to the effect of mutations on the extent of autoprocessing, the pre‐activation process performed by enzymes of the *N*‐terminal nucleophile hydrolase family, of which NylC is a member [32] (see Figure S5). This process and potential enzyme concentration differences due to variable expression levels in the small cultures were both normalized in the treatment of the purified enzymes. While this mutational study represents a showcase of the assay itself, this result also demonstrates that the use of Trylon allows for identification of novel nylon‐degrading enzymes in a rapid assay that is applicable to a more concerted engineering context.

### 2.4 Evaluation of Inactive Variants

A variety of factors may lead to the presentation of false negatives in an enzyme screen. There is, as mentioned above, natural variance in expression levels between different cultures, and protein folding could be impacted by mutations. NylC, being an *N*‐terminal nucleophile hydrolase, introduces an additional complicating factor, where mutations could impact the rate of autoprocessing and, consequently, observed activity. While any nylon hydrolysis assay may correspondingly lead to these false negatives, our assay identifies enzymes that show rapid amide hydrolase activity, and it is natural that low initial rates could be observed due to any or all of these factors. In order to investigate the extent to which Trylon can differentiate between active and inactive enzymes, we chose five inactive enzymes to purify and evaluate for activity against Nylon‐6.

We selected mutants Y146A, Y146M, W192Q, K189Y, and M305T (see Figure S4A–D), alongside NylC_K_TS as a positive control. Proteins were expressed in 25‐mL cultures (see Methods for full details). Analysis of the purified enzymes by SDS‐PAGE (Figure S6A) indicated that mutants Y146A and K189Y did not express, possibly due to mutation‐induced misfolding. The remaining mutants indicate varying states of autoprocessing: whereas NylC_K_TS is almost entirely processed, the inactive precursor (37‐kDa fragment) predominates in the M305T mutant. This is consistent with our previous observation that position 305 is relevant for autoprocessing (Figure S5).

**FIGURE 7:**
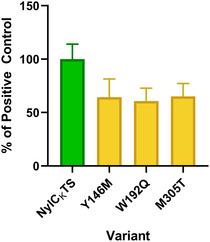
Relative activity of identified inactive mutants from Trylon‐6 screen against Nylon‐6. Values are reported as percentage of the wild‐type activity, and were determined through measurement of total amine content by TNBS, following incubation of Nylon‐6 film (7.9 mg) with enzyme (0.1 mg/mL, 0.25 mL reaction volumes in 20 mM KH_2_PO_4_ buffer, pH 7.3) at 50 °C for 24 h. Values are averages of three replicates, and error bars represent standard error propagated from standard deviation.

To ensure full autoprocessing, each enzyme was incubated at 37 °C for 40 h (Figure S6B); then, activity was measured against Nylon‐6 by TNBS assay. Due to limitations in enzyme quantity, we opted to perform a single endpoint measurement after 24 h. Results are shown in Figure 7.

All the isolable mutants demonstrate partial activity compared to the wild‐type, NylC_K_TS, after 24 h. This result was not entirely surprising, given the numerous important differences between evaluation of fresh lysate and evaluation of purified enzyme, as previously discussed. After normalizing these factors, it is reasonable that single mutations may not completely eliminate activity, especially after an extended reaction time. Furthermore, since this is an endpoint measurement rather than a kinetic evaluation, we cannot conclude the extent to which these mutations affect the initial rate of the reaction. Overall, this result highlights the stringency of the Trylon screen. These enzymes, initially deemed *inactive* following the Trylon‐6 assay, demonstrated only ∼60%–65% the activity of wild‐type NylC_K_TS against Nylon‐6. Conversely, those variants identified as being *active* in the Trylon screen displayed >90% of the wild‐type activity against Nylon‐6 (Figure 6) with the exception of M305V, which showed notable but reduced (∼80%) activity in both the Trylon screen and Nylon assay. This result provides further evidence that the Trylon‐6 assay identifies the most active enzymes that would be carried forward in an extended engineering campaign.

### 2.5 Accelerated Trylon‐6 Endpoint Screen at High Temperature

**FIGURE 8:**
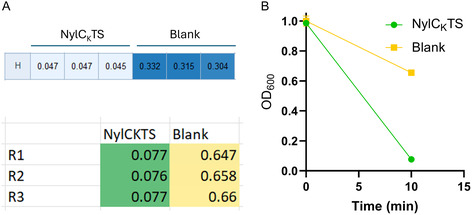
Trylon‐6 assay performed at 65 °C. Reactions contain buffer (20 mM KH_2_PO_4_ pH 7.3, 15% glycerol), BSA (0.1 mg/mL), Trylon‐6 (1 mM), methanol (5% v/v), and enzyme (0.1 mg/mL), with blank reactions containing no enzyme. (A) Endpoint read as visualized by the BioTek Gen5 software (above, raw values) or in Microsoft Excel, using conditional formatting (below, pathlength‐corrected values). (B) Comparison of OD_600_ values before (0 min) and after (10 min) incubation of reaction mixtures at 65 °C.

We have thus far demonstrated the Trylon‐6 assay in so‐called *continuous* mode at 50 °C, which is a temperature most plate readers can maintain. However, as previously mentioned, the NylC‐engineered thermostable enzymes have demonstrated optimal temperatures of ∼70 °C [20]. It is, furthermore, advantageous to screen enzymes under the conditions at which they would be expected to perform, to ensure this activity and thermostability is retained. Therefore, we also adapted the Trylon‐6 activity assay to a *discontinuous* method, allowing for screening at temperatures above the limitations of a plate reader. To do so, wells containing Trylon‐6 and either NylC_K_TS or buffer alone (blank) were incubated at 65 °C for 10 min, after which a single endpoint read was performed at room temperature. Clarification was immediately evident both qualitatively by visual inspection of the plate and quantitatively by endpoint read (Figure 8A) and when compared to a pre‐incubation read (Figure 8B). Although a moderate decrease in OD_600_ is observed in the blank due to temperature‐induced partial solubilization, the difference in the post‐incubation OD_600_ values is obvious when comparing the blank and enzyme‐containing reactions. While some plate reader software colour‐codes OD values automatically, we propose that the conditional formatting tool on Microsoft Excel could also be applied to aid in rapid visualization of wells containing active enzyme.

## 3 Conclusions

We designed nylon surrogate substrates (Trylons) and applied them in a rapid, continuous assay capable of quantifying amidase activity in nylon‐degrading enzymes. Evaluation of the substrates revealed unprecedented selectivity in the orientation of amide bonds required to be recognized by NylC enzymes. We demonstrated that Trylon‐6 and Trylon‐66 are hydrolysed by NylCs following a trend similar to their respective polymer analogues, Nylon‐6 and Nylon‐66, suggesting that this activity may predict nylon hydrolysis activity in NylC variants. We used the Trylon assay to screen four mutant libraries of NylC and identified variants that were active against Nylon‐6. We envision the Trylon assay will prove useful for the rapid, preliminary identification of active NylC variants. When performed in continuous mode, a plate can be evaluated in less than 20 min, where initial slopes are evident in under 5 min. Conversely, when performed in endpoint mode following a 10‐min incubation at 65 °C, the throughput of this assay is limited only by the rate at which the user’s instrument can read a plate. Thus, a thousand samples could be screened, from lysate to activity data, in well under an hour, eliminating all but the best candidates. Then, targeted screening of known active variants could be performed, saving preprocessing time, expensive standardized nylon substrates and derivatization agents, or analytical chromatography resources. We propose that this assay may be used in future engineering efforts to accelerate the identification of new and improved nylon‐hydrolysing enzymes.

## 4 Materials and Methods

### 4.1 General Remarks

All reagents and solvents were purchased from commercial sources and used without further purification. Thin‐layer chromatography (TLC) was performed using silica plates. ^1^H‐ and ^13^C‐NMR spectra were recorded on a Bruker 400‐MHz spectrometer, and chemical shifts were reported in ppm, referenced to the deuterated solvent peak. High‐resolution mass spectra were obtained with a quadrupole time‐of‐flight (QTOF) analyser and electrospray ionization (ESI). SDS‐PAGE was performed using Bio‐Rad Mini‐Protean TGX pre‐cast, stain‐free gels (4%–20% polyacrylamide) and visualized using a Bio‐Rad ChemiDoc MP imager or with Coomassie staining. Agarose gels were prepared immediately prior to use and contained 1% agarose (w/v) and 1 μg/mL ethidium bromide. DNA was separated on agarose gel at 105 V in Tris‐acetate‐EDTA (TAE) buffer and visualized using a Bio‐Rad ChemiDoc MP imager.

### 4.2 Synthesis Methods

Synthetic methods and characterization data may be found in the Supporting Information.

### 4.3 Library Generation by Site‐Saturation Mutagenesis

For each of the four positions in the gene NylC_K_TS selected for mutation, forward and reverse primers were designed, where the forward primer contained the degenerate codon NNK at the 5′‐end (see Table S3). Full‐plasmid PCR was performed using Q5 High‐Fidelity DNA polymerase (New England Biolabs) and NylCK‐TS_pET28a as the template. Following amplification of the gene as confirmed by agarose gel electrophoresis, the DNA was purified (E.Z.N.A. Cycle Pure Kit, Omega BioTek); then, a digest of the template was performed using DpnI (New England Biolabs). Following digestion, the amplicon was purified again using the Cycle Pure Kit, then phosphorylated (T4 PNK, New England Biolabs), and ligated (T4 DNA Ligase, New England Biolabs). The circularized amplicon was then transformed into chemically competent *E. coli* BL21‐DE3 cells and selected on LB agar plates supplemented with 50 μg/mL kanamycin.

### 4.4 Enzyme Expression and Purification

#### 4.4.1 Small‐Scale Expression of Mutant Enzymes and Preparation of Cell Lysate for Screening

Colonies (90 per plate) were selected from *E. coli* BL21‐DE3 cells transformed with each NylC_K_TS site‐saturation library (1 library = 1 mutant position) and used to inoculate 1‐mL cultures containing LB‐kanamycin in a deep‐well 96‐well plate (Axygen) (the *reference plate*). Plates also contained duplicate control wells, either inoculated with colonies transformed with the wild‐type plasmid (NylCK‐TS_pET28a) or with empty vector (pET28a), or containing no bacteria (sterility control). Plates were sealed and shaken overnight at 37 °C. Then, 200 μL of each pre‐culture was used to inoculate 1‐mL expression cultures containing 2×YT media supplemented with kanamycin (50 μg/mL), in a deep‐well 96‐well plate (*expression plate*). To each well of the reference plate was added 1 mL of sterile LB media containing glycerol (60% w/w). The cultures were mixed by pipetting; then, the reference plates were stored at −80 °C.

The expression plates were shaken at 37 °C for 4 h and then induced through addition of IPTG to each 1‐mL culture, to a final concentration of 0.1 mM. The plates were then shaken at room temperature for 18 h. Cells were pelleted by centrifugation of the plates at 2700 × g for 20 min at 4 °C. The pellets were resuspended in 40 μL lysis buffer (50 mM Tris pH 7.5, 10 mM imidazole, 100 mM NaCl, containing 0.1 mg/mL lysozyme, and 10 μg/mL DNase). The cells were lysed by eight freeze–thaw cycles (alternating between −78 °C and 40 °C baths). The lysates were then clarified by centrifugation at 2700 × g for 1 h and used immediately.

#### 4.4.2 Preparative Expression and Purification of NylCs

Overexpression of NylC enzymes was performed following the protocol published by Bell et al. [20] with certain modifications. In summary, pET28a vectors containing the corresponding NylC gene were obtained from AddGene (see Table S1 for full plasmid details), transformed into BL21‐DE3 *E*. *coli* cells, and selected on LB agar plates supplemented with 50 μg/mL kanamycin. Single colonies were picked to prepare liquid small cultures, which served to inoculate 2 × YT medium expression cultures supplemented with 50 µg/mL of kanamycin. Those cultures were incubated at 37 °C to an OD of 1; then, 0.1 mM IPTG was added to induce expression with shaking for 20 h at 20 °C. To purify the enzymes, cells were collected by centrifugation at 3400 × g for 20 min at 4 °C. The cells were then resuspended (1:50 v/v) in lysis buffer (Tris 50 mM pH 7.5, 10 mM imidazole, 100 mM NaCl, 1 mg/mL of lysozyme, and 10 μg/mL of DNase) and lysed by sonication (Hielscher Ultrasonic Processor UP50H) at 70% amplitude using three rounds of a 3:2 s on and off cycle for 1 min followed by 1 min on ice. The soluble and insoluble fractions were separated by centrifugation at 19,300 × g for 15 min at 4 °C. The soluble fraction was incubated with His 60 Ni Superflow resin (1:1000 v/v resin:culture) at 4 °C for 60 min on a rotary shaker. Bound proteins were then washed with 4 × 10 column volumes of lysis buffer (without lysozyme or DNase) and eluted using 3 × 10 column volumes of elution buffer (Tris 50 mM pH 7.5, 300 mM imidazole, and 300 mM NaCl). Collected fractions were analysed by SDS‐PAGE, and fractions containing pure protein were concentrated using 10‐kDa MWCO Amicon tubes (Sigma Aldrich) and then buffer‐exchanged with 3 × 15 mL storage buffer (20 mM potassium phosphate pH 7.3 and 10% glycerol). Protein samples were incubated at 37 °C for 16 h and then centrifuged prior to quantification via Bradford assay. Purified protein aliquots were flash‐frozen and stored at −78 °C until use.

#### 4.4.3 Preparative Expression and Purification of Inactive NylC Mutants

Expression and purification of mutants Y146A, Y146M, W192Q, K189Y, and M305T were performed alongside NylC_K_TS as described above, with a few modifications. Precultures were inoculated from glycerol stocks (from the reference plate). Overexpression of protein was performed in 25‐mL cultures, and due to the small volume of the corresponding lysate, lysis was performed by freeze–thaw (eight cycles alternating between −78 °C and 40 °C). Following purification and buffer exchange, samples were incubated for 40 h at 37 °C prior to quantification.

### 4.5 General Procedure for the Hydrolysis of Diamide Substrates by NylC Enzymes (GP1)

Stock solutions of each reaction component were prepared as follows: potassium phosphate buffer (200 mM, pH 7.3), glycerol (50% w/w in H_2_O), BSA (2 mg/mL), substrate (20 mM in MeOH), and enzyme (1 mg/mL). A master mix was prepared containing all components except enzyme, such that each 200‐μL reaction solution would contain phosphate buffer (20 mM), glycerol (15% v/v), BSA (0.1 mg/mL), and substrate (1 mM, 5% MeOH). The mixture was vortexed to produce a homogeneous suspension, and 180 μL was aliquoted in 96‐well plates. The plates were incubated at 50 °C for 25 min to allow the optical density (OD_600_) to equilibrate. The reactions were then initiated by addition of 20 μL of enzyme solution (to a final concentration of 0.1 mg/mL); then, OD_600_ was monitored over time using a BioTek Synergy H1 plate reader. Reactions were conducted in triplicate, and reaction rates were determined by linear regression of the initial slopes derived from the plot of OD_600_ over time. Rates were converted to units of μM/s using calibration curves prepared for each substrate (see Figure S1) and corrected by subtraction of rates from blank reactions of the corresponding substrate containing no enzyme.

#### 4.5.1 Lysate‐Mediated Hydrolysis of Compound 1

Reaction mixtures were prepared in 96‐well plates as described in GP1, except that the reactions were initiated through addition of 10% v/v of clarified lysate. Reactions were monitored at 600 nm for 1 h, or manually terminated if reactions were complete prior to that time. All reactions with wild‐type enzyme were complete after 20 min.

#### 4.5.2 Hydrolysis of Compound 1 Monitored by Discontinuous Assay

Reaction mixtures were prepared in 96‐well plates as described in GP1, containing all components except enzyme. Enzyme (to a final concentration of 0.1 mg/mL) or buffer (no enzyme blank) was added to each well; then, an endpoint absorbance measurement was immediately taken at 600 nm. The plate was incubated at 65 °C for 10 min; then, a second absorbance measurement was acquired. Reactions were performed in triplicate for each condition (enzyme or blank).

### 4.6 Sample Preparation and Product Analysis by UPLC

Samples were obtained from hydrolysis reactions following GP1 for a reaction of 90 min and then prepared for UPLC analysis by diluting 1:1 in acetonitrile supplemented with 0.1% trifluoroacetic acid. Samples were then centrifuged for 5 min at 21,300 × g, and the supernatants were collected for analysis. Samples were analysed by UPLC (Waters AQUITY UPLC coupled with the Waters MassLynx Mass Spectrometer). Separation was achieved at a flow rate of 0.3 mL/min over 23 min with a mobile phase consisting of a gradient of 10%–90% acetonitrile in water supplemented with 10% formic acid. Mass data were collected in ESI + mode, and quantification of reaction components was determined from integration of extracted ion chromatograms for each species [17].

### 4.7 Nylon Film Hydrolysis

Nylon‐6 film (0.2 mm thickness) and Nylon‐66 (0.5 mm thickness) were purchased from Goodfellow. Standard‐size disks were created using a 6.5‐mm‐diameter hole puncher to produce samples of 8.2 mg or 18.1 mg for Nylon‐6 and Nylon‐66, respectively. Reactions were performed in 1‐mL reaction volumes and contained potassium phosphate buffer (20 mM, pH 7.3), enzyme (0.1 mg/mL), and nylon film (Nylon‐6, 8.2 mg; Nylon‐66, 18.1 mg). Reaction mixtures containing all components except enzyme were heated to 50 °C for 5 min, followed by initiation of the reaction through addition of enzyme. Reactions were incubated at 50 °C for 24 h, and 80‐μL aliquots were acquired at various timepoints and stored at −20 °C until quantification by trinitrobenzenesulfonate (TNBS). Blank reactions were performed containing all components except enzyme, and rates were corrected by subtracting the rate of uncatalysed nylon degradation. Rates represent a corrected average of reactions performed in duplicate or triplicate, as described.

### 4.8 Quantification of Liberated Amines by TNBS

TNBS was purchased from Sigma Aldrich as a 5% solution in H_2_O. The TNBS stock was diluted 360‐fold in sodium bicarbonate buffer (0.5 M, pH 8.5). In a 96‐well plate, the TNBS solution (160 μL) was added to 40 μL of sample. Calibration curves were prepared on each 96‐well plate alongside the samples, by diluting 40 μL of aminohexanoic acid dimer (0–500 μM in 20 mM potassium phosphate buffer, pH 7.3) with TNBS solution (160 μL). The plate was incubated at 37 °C for 2 h and then carefully sonicated to liberate any bubbles produced. Absorbance was measured at 335 nm and converted to concentration using the slope of the calibration curve.

### 4.9 Crystallinity Determination by Differential Scanning Calorimetry

Crystallinity was determined using a TA instruments Discovery 2500 differential scanning calorimeter. Samples (3–6 mg) were added to aluminium pans that were hermetically sealed and then pierced to produce a pinhole. Samples were heated from 0 °C to 250 °C or 0 °C to 300 °C for Nylon‐6 or Nylon‐66, respectively, at a ramp rate of 10°C per minute. Crystallinity was determined by integration of the melt thermogram and expressed as a percentage based on theoretical melting enthalpies of 100% crystalline samples (230.1 J/g for Nylon‐6; 255.8 J/g for Nylon‐66). Measurements were performed in triplicate for each polymer.

## Funding

This study was supported by the Natural Sciences and Engineering Research Council of Canada (RGPIN‐2025‐06384).

## Conflicts of Interest

The authors declare no conflicts of interest.

## Supporting information

The following additional information can be found in the Supporting Information: synthetic methods, supplemental tables and figures, and characterization data. The authors have cited additional references within the Supporting Information [33, 35].

## Data Availability

The data that supports the findings of this study are available in the supplementary material of this article.
