# Supplementary material for: Trylons: Polyamide Surrogate Substrates Enable High‐Throughput Screening of Nylon‐Degrading Enzymes
Source: Chembiochem. 2026 Jul 8;27(13):e70459. doi: 10.1002/cbic.70459 (PMC13343204; doi:10.1002/cbic.70459)
Supplement: Supplementary file 1 — The following additional information can be found in the Supporting Information: synthetic methods, supplemental tables and figures, and characterization data. The authors have cited additional references within the Supporting Information [33, 35]. [file CBIC-27-e70459-s001.pdf]

Supporting Information for

Trylons: Polyamide Surrogate Substrates Enable High-Throughput Screening of Nylon-Degrading Enzymes

Alana M. M. Rangaswamy,[1] Francis M. Roy,[1] Maria E. Cleveland,[2] and Jeffrey W. Keillor*[1]

[1]Department of Chemistry and Biomolecular Sciences, University of Ottawa, 10 Marie Curie Pvt, Ottawa, ON K1N 6N5 (Canada)

[2] Department of Chemistry, Queen's University, 90 Bader Ln, Kingston, ON, K7L 3N6 (Canada)

*Corresponding Author: jkeillor@uottawa.ca

Table of Contents

Synthetic methods **.......... 2**

Supplemental Tables and Figures **.......... 7**

Characterization Data **.......... 13**

References **.......... 19**

Synthetic methods

General procedure 2 (GP2) for amide bond formation

A solution of acid (1.1 equiv.), HBTU (1.6 equiv.), and DIPEA (3 equiv.) in DCM (0.16 M with respect to the limiting reagent) was stirred at R.T. for 30 minutes, following which amine (1 equiv.) was added. The reaction was monitored by TLC (10% MeOH, 45% DCM, 45% hexanes) and visualized with ninhydrin stain. Following completion of the reaction, the reaction mixture was evaporated in vacuo, then the residue was purified as specified.

***N*-Boc-aminohexanoic acid**

The title compound was prepared as previously described.[1]

***N*-hexyl-6-(*N*-Boc)aminohexanamide (4)**

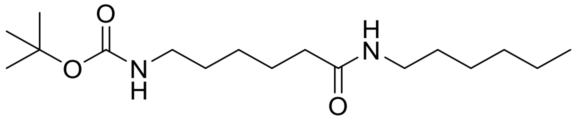

Compound **4** was synthesized following **GP2** with 200 mg (1.965 mmol) of hexylamine, and *N*-Boc-aminohexanoic acid as the coupling partner. Following evaporation of the reaction mixture, the residue was redissolved in EtOAc (~20 mL), then washed with equal volumes of 10% AcOH, brine, saturated $NaHCO_3$ (3 ×) and a final wash with brine. The organic phase was dried over $MgSO_4$, then evaporated to give a viscous oil, which turned into to a white solid after drying overnight. The solid was then resuspended in hexanes, sonicated to give a fine suspension, then collected by filtration. The product was dried to give a flaky white solid (587 mg, 95%). ^{1}H NMR (400 MHz, $CDCl_3$) δ 5.48 (s, 1H), 4.55 (s, 1H), 3.22 (q, *J* = 6.7 Hz, 2H), 3.10 (q, *J* = 6.4 Hz, 2H), 2.15 (t, *J* = 7.5 Hz, 2H), 1.64 (qu., *J* = 7.6 Hz, 2H), 1.48 (m, 4H), 1.43 (s, 9H), 1.31 (m, 8H), 0.87 (t, *J* = 6.7 Hz, 3H). ^{13}C NMR (101 MHz, $CDCl_3$) δ 172.9, 156.2, 79.2, 40.5, 39.7, 36.8, 31.6, 29.9, 29.8, 28.5, 26.7, 26.5, 25.5, 22.7, 14.1. HRMS (ESI) calc'd for $C_{17}H_{34}N_2O_3Na$ ($[MNa]^+$): 337.2467, found: 337.2468.

N-hexyl-6-aminohexanamide hydrochloride (1b)

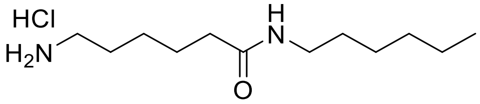

To a solution of **4** (500 mg, 1.59 mmol) in DCM (8 mL) was added a solution of HCl (4 M in dioxane, 8 mL). The reaction was monitored by TLC (10% MeOH, 45% DCM, 45% hexanes) and visualized with ninhydrin stain. Upon completion, the reaction was evaporated in vacuo, then the residue was suspended in hexane and triturated with sonication. The hexane was decanted off and the product dried under high vacuum. The product was isolated in quantitative yield (400 mg). ^{1}H NMR (400 MHz, DMSO) δ 7.83 (m, 4H), 2.99 (q, J = 6.5 Hz, 2H), 2.73 (s, 2H), 2.04 (t, J = 7.4 Hz, 2H), 1.49 (m, 4H), 1.35 (m, 2H), 1.22 (m, 8H), 0.84 (t, J = 6.8 Hz, 3H). ^{13}C NMR (101 MHz, DMSO) δ 172.2, 38.9, 38.6, 35.3, 31.1, 29.2, 26.9, 26.2, 25.6, 25.0, 22.2, 14.1. HRMS (ESI) calc'd for $C_{12}H_{27}N_2O$ ([MH]$^{+}$): 215.2123, found: 215.2123.

N-hexyl-6-(*N*-hexanoyl)aminohexanamide (1)

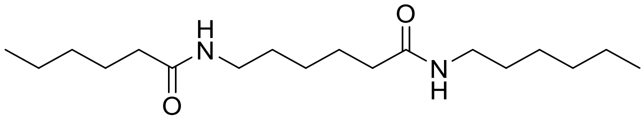

The reaction proceeded following **GP2** with 70 mg (0.279 mmol) of amine **1b** and hexanoic acid as the coupling partner. Following evaporation of the reaction mixture, the residue was redissolved in 2-methyl-THF (10 mL), then washed with an equal volume of 10 % AcOH, then brine, then 3 × 10 mL saturated $NaHCO_3$, then brine. The organic phase was dried over $MgSO_4$ and evaporated to give a white solid, which was triturated with a solution of 1:1 hexanes: ethyl acetate, then filtered to give a white powder (58 mg, 67%). ^{1}H NMR (400 MHz, MeOD) δ 3.15 (dt, J = 2.3, 7.1 Hz, 4H), 2.17 (q, J = 7.0 Hz, 4H), 1.61 (q, J = 7.5 Hz, 4H), 1.50 (q, J = 7.5 Hz, 4H), 1.32 (m, 12H), 0.91 (m, 6H).^{13}C NMR (101 MHz, MeOD) δ 176.3, 176.0, 40.4, 40.2, 37.1, 37.0, 32.7, 32.5, 30.4, 30.1, 27.7, 27.5, 26.8, 26.7, 23.6, 23.4, 14.4, 14.3. HRMS (ESI) calc'd for $C_{18}H_{36}N_2O_2Na$ ([MNa]$^{+}$): 335.2674, found: 335.2649.

Hexanedioic acid bis-hexylamide (2)

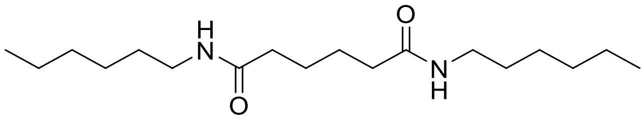

To a stirred solution of hexylamine (261 µL, 1.976 mmol) and DIPEA (494 µL, 2.823 mmol) in Et_2O (4 mL) was added adipoyl chloride (137 µL, 0.941 mmol) dropwise over ~1 minute. Immediately following addition of the diacyl chloride, a large amount of white precipitate formed. The suspension was diluted with 15 mL of Et_2O, then the precipitate collected by vacuum filtration. The solid was resuspended in H_2O (aided by sonication), then collected by vacuum filtration. The resulting solid was washed with acetone and dried to give the product as a white powder (133 mg, 45%). ^{1}H NMR (400 MHz, $CDCl_3$) δ 5.78 (br. s, 2H), 3.22 (q, J = 7.0 Hz, 4H), 2.18 (m, 4H), 1.65 (m, 4H), 1.49 (m, 4H), 1.29 (m, 12H), 0.87 (t, J = 6.8 Hz, 6H). Characterization data are consistent with literature.[2]

(*N*,*N′*-bis-hexanoyl)-1,6-hexanediamine (3)

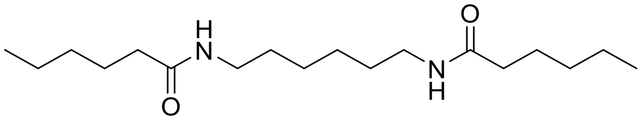

To a stirred solution of hexanoic acid (237 µL, 1.893 mmol) and DMF (2 drops) in DCM (2 mL) was added oxalyl chloride (175 µL 2.066 mmol) under a stream of nitrogen. The consumption of oxalyl chloride was indicated by the evolution of gas, and the reaction was stirred until no more bubbles were formed (~5 minutes). In a separate vessel was stirred 1,6-hexanediamine (120 µL, 0.861 mmol) and DIPEA (450 µL, 2.583 mmol) in DCM (2 mL). To the solution of diamine was added the solution of hexanoyl chloride dropwise over ~2 minutes. Immediately following addition of the acyl chloride, a large amount of white precipitate formed. The suspension was diluted with 15 mL of Et_2O, and the solid collected by vacuum filtration. The solid was washed with water (20 mL) and acetone (20 mL), and dried on vacuum to give the product as a fluffy white solid (143 mg, 55%). ^{1}H NMR (400 MHz, $CDCl_3$) δ 5.58 (br. s, 2H), 3.24 (q, J = 6.6 Hz, 4H), 2.16 (t, J = 7.6 Hz, 4H), 1.64 (m, 4H), 1.49 (m, 4H), 1.31 (m, 12H), 0.89 (t, J = 6.8 Hz, 6H). Characterization data are consistent with literature.[3]

Methyl (*N*-hexanoyl)-6-aminohexanoate (5)

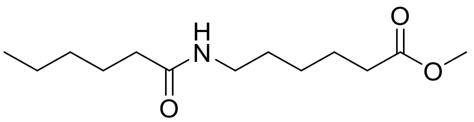

The reaction proceeded following **GP2** with 200 mg (1.100 mmol) of methyl-6-aminohexanoate hydrocholoride. The reaction was complete after 18 hours. Following evaporation of the reaction mixture, the residue was redissolved in ethyl acetate (20 mL), then washed with 3 × 10 mL of 10 % AcOH, then brine, then 3 × 10 mL saturated $NaHCO_3$, then brine. The organic phase was dried over $MgSO_4$ and concentrated to give a colourless residue. The crude product was adsorbed onto silica, washed with 1:1 hexanes : DCM, then eluted with 9:1 DCM : MeOH. The fraction containing the product was evaporated in vacuo and dried under high vacuum to give the product as a colourless oil (158 mg, 59%). ^{1}H NMR (400 MHz, $CDCl_3$) δ 5.53 (br. s, 1H), 3.66 (s, 3H), 3.24 (q, *J* = 5.0 Hz, 2H), 2.31 (t, *J* = 7.4 Hz, 2H), 2.14 (t, *J* = 7.6 Hz, 2H), 1.63 (m, 4H), 1.50 (qu, *J* = 7.4 Hz, 2H), 1.32 (m, 6H), 0.88 (t, *J* = 6.9 Hz, 3H). ^{13}C NMR (101 MHz, $CDCl_3$) δ 174.2, 173.4, 51.6, 39.3, 37.0, 34.0, 31.6, 29.4, 26.5, 25.6, 24.6, 22.5, 14.1. HRMS (ESI) calc'd for $C_{13}H_{25}NO_3Na$ ($[MNa]^+$): 266.1732, found: 266.1759.

***N*-hexanoyl-6-aminohexanoic acid (1a)**

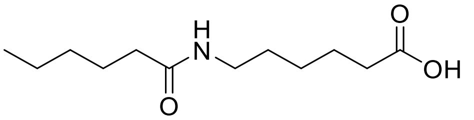

Compound **5** was dissolved in MeOH (3 mL) and aqueous NaOH (2 M, 3 mL) and the reaction was stirred for 48 hours at R.T. The reaction mixture was concentrated *in vacuo* to remove the methanol, then diluted in H_2O (10 mL) and washed with Et_2O (10 mL). The aqueous phase was acidified through dropwise addition of 12 M HCl to a pH of ~1, then extracted with 3 × 10 mL EtOAc, The EtOAc extracts were combined and washed with 10 mL of brine, then dried over $MgSO_4$ and evaporated to give a clear residue, which was dried under vacuum to produce flaky white crystals (125 mg, 89%). ^{1}H NMR (400 MHz, DMSO) δ 7.75 (t, *J* = 5.4 Hz, 1H), 2.99 (q, *J* = 6.5 Hz, 2H), 2.17 (t, *J* = 7.4 Hz, 2H), 2.01 (t, *J* = 7.4 Hz, 2H), 1.46 (m, 4H), 1.36 (qu, *J* = 7.2 Hz, 2H), 1.23 (m, 6H), 0.84 (t, *J* = 7.1 Hz, 3H). ^{13}C NMR (101 MHz, DMSO) δ 174.4, 172.0, 38.1,

35.3, 33.5, 30.8, 28.7, 25.8, 24.9, 24.1, 21.7, 13.8. HRMS (ESI) calc'd for $C_{12}H_{23}NO_3Na$ ($[MNa]^+$): 252.1576, found: 252.1594.

Supplemental Tables and Figures

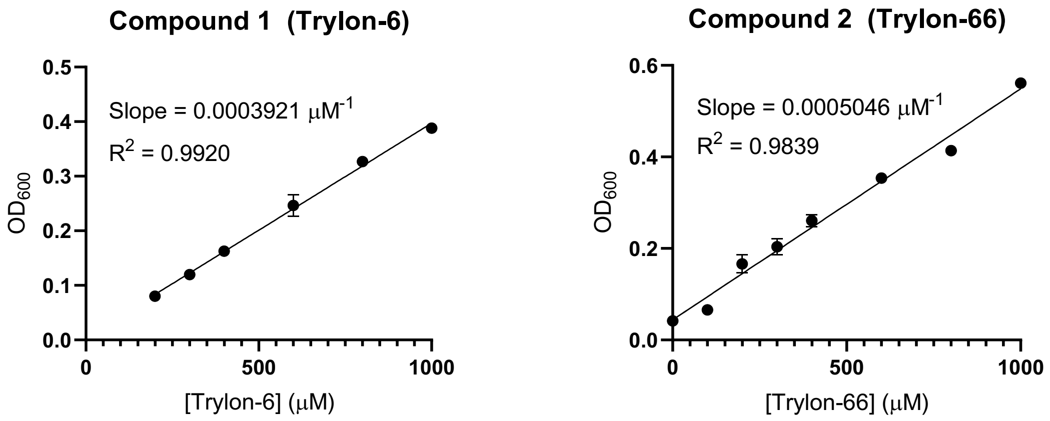

Figure S1. Calibration curves for Trylon-6, and Trylon-66 measured at 600 nm on a BioTek plate reader, at 50 °C. Standard suspensions contain: substrate (0-1000 μM), potassium phosphate buffer (20 mM, pH 7.3), glycerol (15%), BSA (0.1 mg/mL), MeOH (5%). Data points are averages of three replicates, and error bars show standard deviations

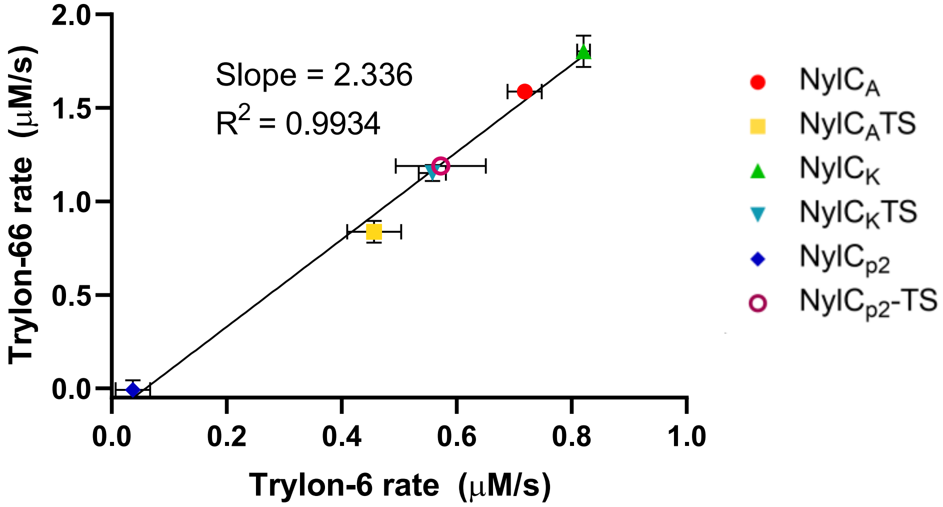

Figure S2. Correlation of rates for the hydrolysis of Trylon-6 or Trylon-66 by NylC enzymes. Each datapoint represents a different enzyme (see Figure 3 in the main text). Error bars represent the standard deviation of triplicates.

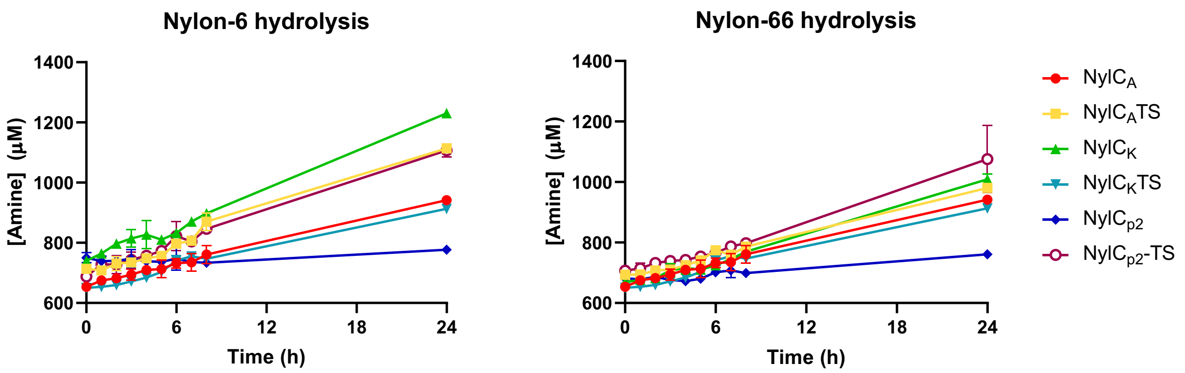

Figure S3. Concentration of amines produced over time, as quantified by TNBS, by reaction of each NylC enzyme with either Nylon-6 or Nylon-66 film. Reaction conditions: NylC (0.1 mg/mL), potassium phosphate buffer (20 mM, pH 7.3), nylon film (Nylon-6: 8.2 mg, Nylon-66: 18.1 mg), 50 °C. Values for nylon hydrolysis are averages of two replicates, with error bars showing standard deviations.

Table S1. Initial rates of Nylon-6 and Nylon-66 hydrolysis by NylC enzymes. Values are averages of duplicate experiments, with errors shown in parentheses.

Enzyme	Nylon-6 hydrolysis rate (µM/h)	Nylon-66 hydrolysis rate (µM/h)	Relative rate of Nylon-66 hydrolysis (% of Nylon-6 rate)
**NylC$_A$**	17.9 (2.2)	11.9 (0.6)	66
**NylC$_A$TS**	16.0 (0.7)	11.0 (0.9)	69
**NylC$_K$**	20.0 (0.8)	14.4 (1.2)	72
**NylC$_K$TS**	15.7 (0.9)	11.5 (0.4)	73
**NylC$_{p2}$**	1.3 (0.6)	3.6 (0.2)	290
**NylC$_{p2}$TS**	17.1 (2.3)	15.4 (5.1)	91

Table S2. Plasmids acquired from AddGene for the expression of NylC variants.

Enzyme	Plasmid Name	Addgene ID
**NylC$_A$**	NylCA_pET28a	215418
**NylC$_A$-TS**	NylCA-TS_pET28a	215419
**NylC$_{p2}$**	NylCp2_pET28a	215420
**NylC$_{p2}$-TS**	NylCp2-TS_pET28a	215421
**NylC$_K$**	NylCK_pET28a	215422
**NylC$_K$-TS**	NylCK-TS_pET28a	215423

Table S3. Primers used for site-saturation mutagenesis at positions 146, 192, 189, and 305. The letters F and R indicate the forward and reverse primers at each position.

Primer	Sequence
NylC_Y146X-F	5′ – NNKGACTTCTCTGCCCGTTCAAC – 3′
NylC_Y146X-R	5′ – GATTACTGCGGAAGACACCAGTTG – 3′
NylC_W192X_F	5′ – NNKGATCGTACCGAGATCACAGGT – 3′
NylC_W192X_R	5′ – ATCAACTTTGCCCGCGCTG – 3′
NylC_K189X_F	5′ – NNKGTTGATTGGGACCGTACCGAG – 3′
NylC_K189X_R	5′ – GCCTGCGCTTGCGCTCAT – 3′
NylC_M305X_F	5′ – NNKGACGGCGATACCCTGTTC – 3′
NylC_M305X_R	5′ – ATCGGTGTGAAATGGCTGGATACC – 3′

Figure S4. Bitmaps for saturation mutagenesis screens showing OD_{600} vs time curves in each well. Sequencing results for selected wells are shown as one-letter codes superimposed on the corresponding well. **Control wells:** A1, F12: Wild-type (NylC$_K$TS); B1, G12: Empty vector; C1, H12: no cells control.

a) Y146X:

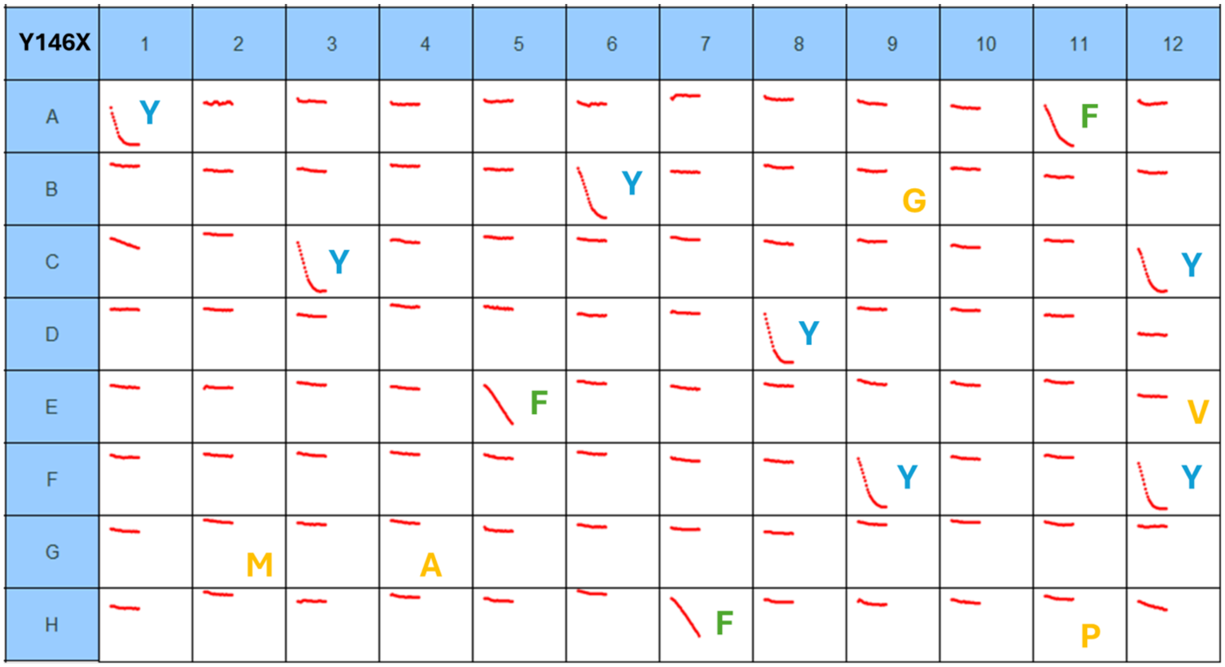

b) W192X:

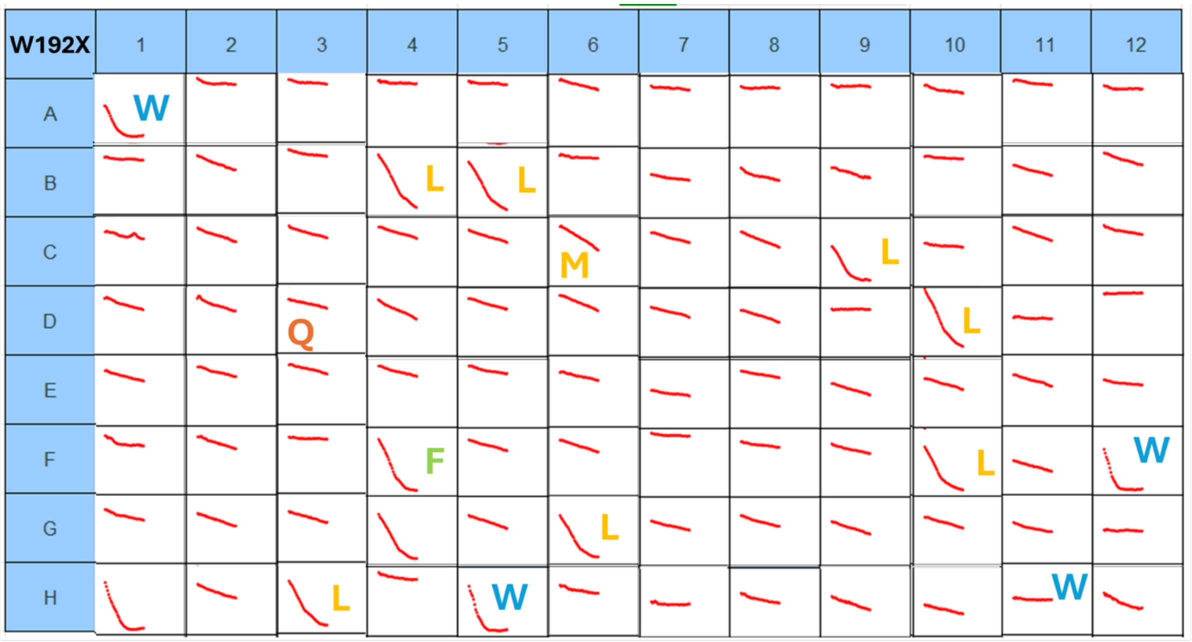

c) K189X:

K189X	1	2	3	4	5	6	7	8	9	10	11	12
A	K			R				K				
B										Y		
C		K					K					K
D					A							
E									K			
F	K											K
G								K				
H	G			K						K		

d) M305X:

M305X	1	2	3	4	5	6	7	8	9	10	11	12
A	M					L				M		
B			L									
C					T	L						
D	C							H				
E				G							L	L
F									Q			M
G		C				T						
H					V						R	

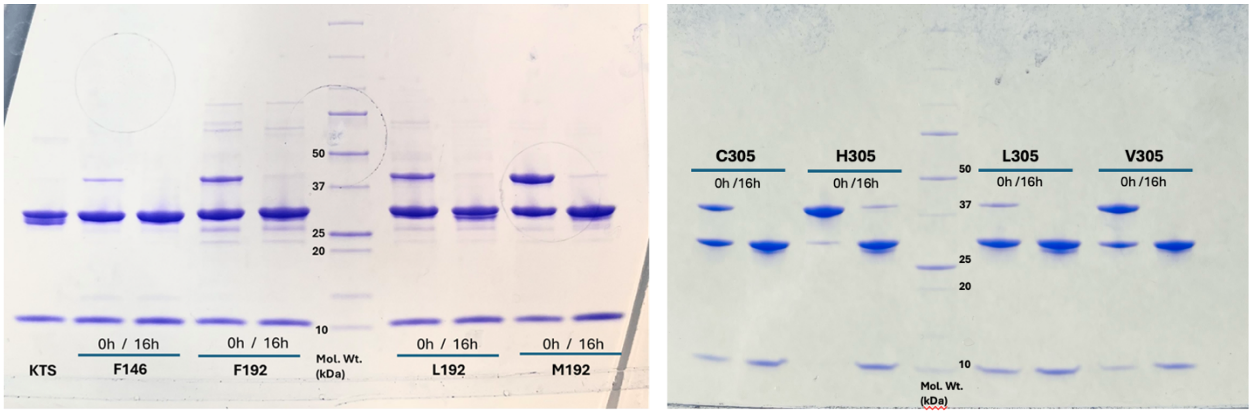

Figure S5. Analysis of each active NylC$_K$TS variant by SDS-PAGE following Ni-affinity purification, after incubation at 37 °C for 0 h or 16 h. The inactive precursor appears as a band at ~37 kDa, whereas the active protein appears as two bands at 27 and 10 kDa.

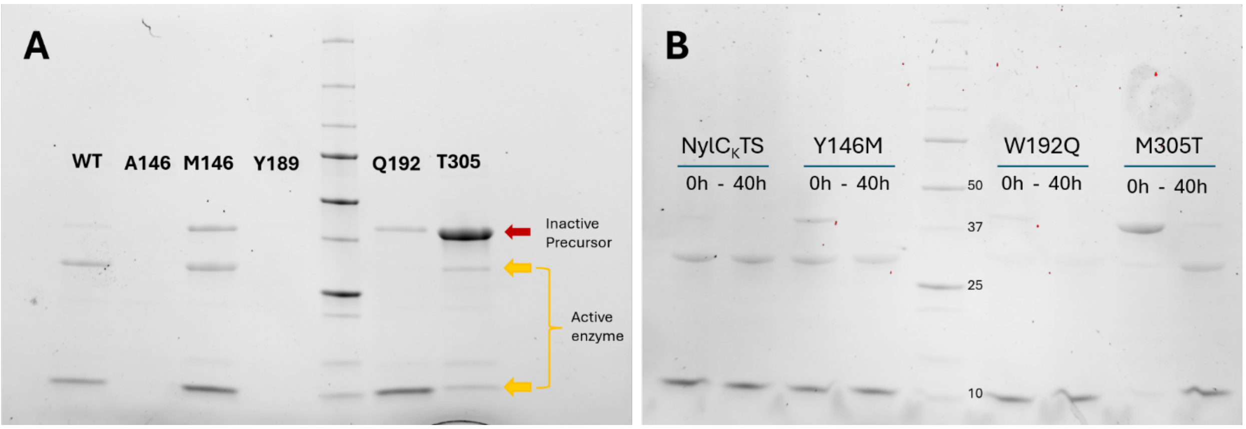

Figure S6. SDS-PAGE analysis of variants identified as inactive by Trylon-6 screen, following Ni-affinity purification. A) Elution fractions of all 5 purified inactive enzymes and NylC$_K$TS (expressed concurrently as a positive control), where variants Y146A and K189Y did not express. B) Purified enzyme (0.5 mg/mL) for all expressing inactive variants, before (0 h) and after (40 h) incubation at 37 °C to promote autoprocessing.

Characterization Data

NMR spectra of all new synthesized compounds

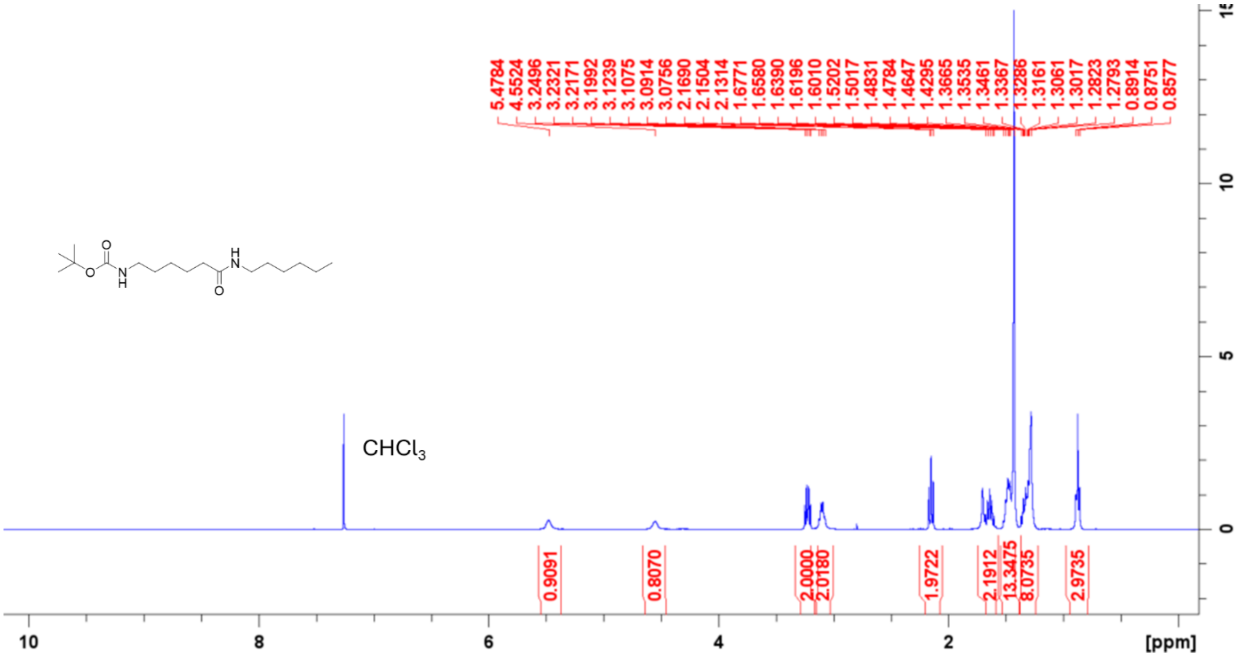

Figure S7. ^{1}H NMR spectrum (400 MHz, $CDCl_3$) of 4.

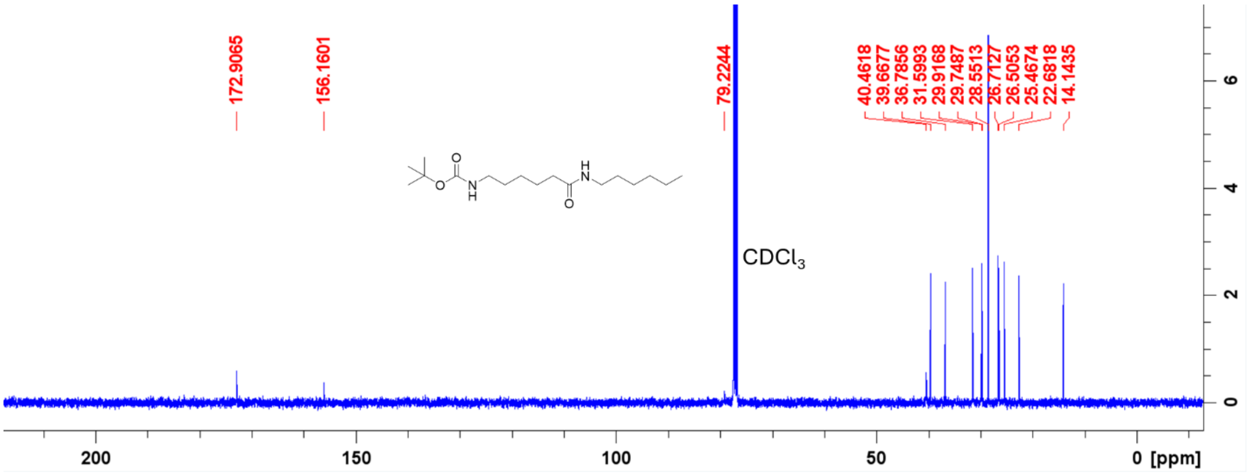

Figure S8. ^{13}C NMR spectrum (101 MHz, $CDCl_3$) of 4.

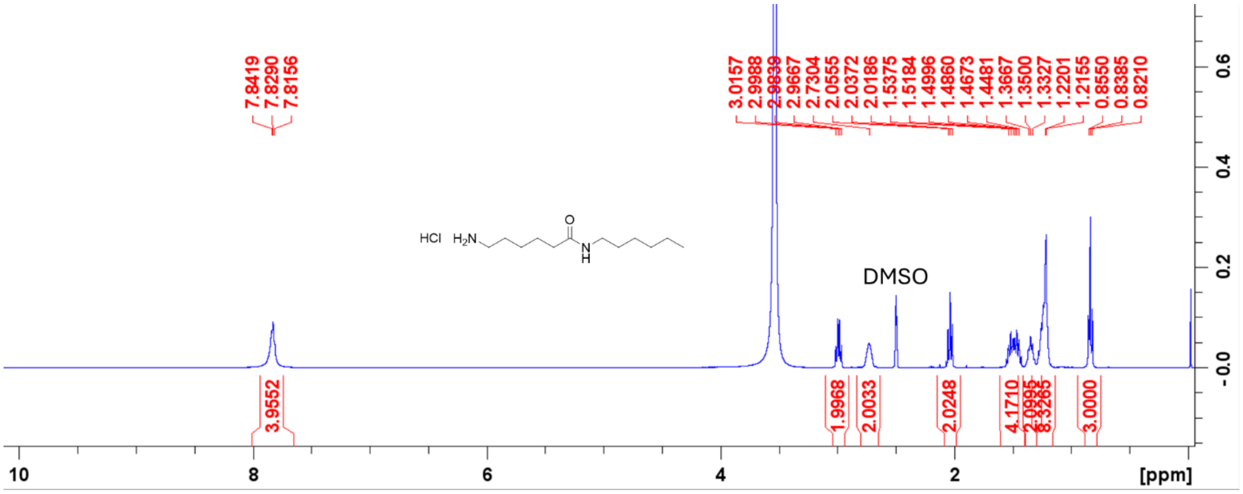

Figure S9. ^{1}**H NMR spectrum (400 MHz, d$_6$-DMSO) of 1b.**

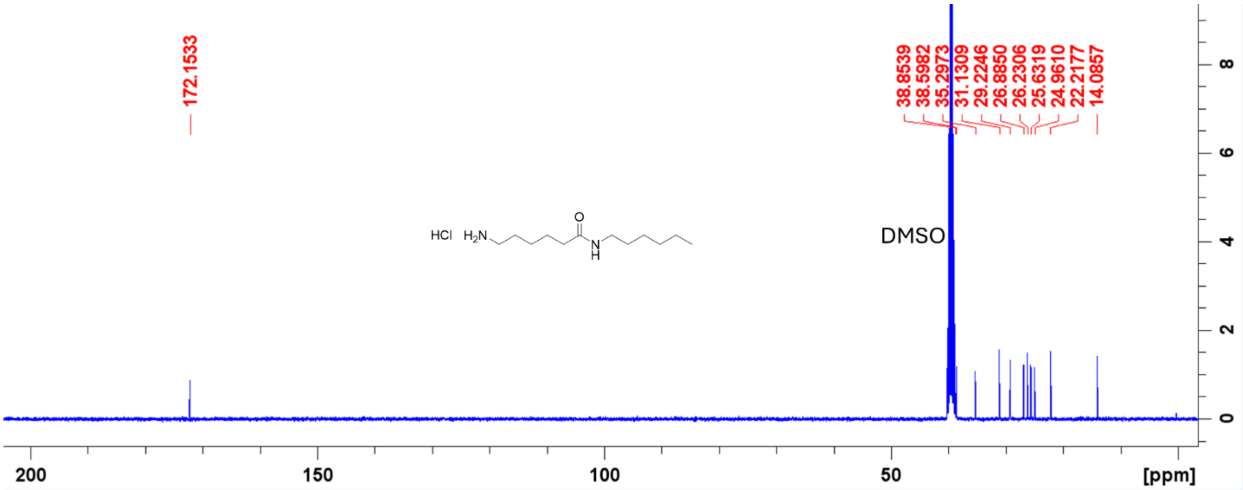

Figure S10. ^{13}**C NMR spectrum (101 MHz, d$_6$-DMSO) of 1b.**

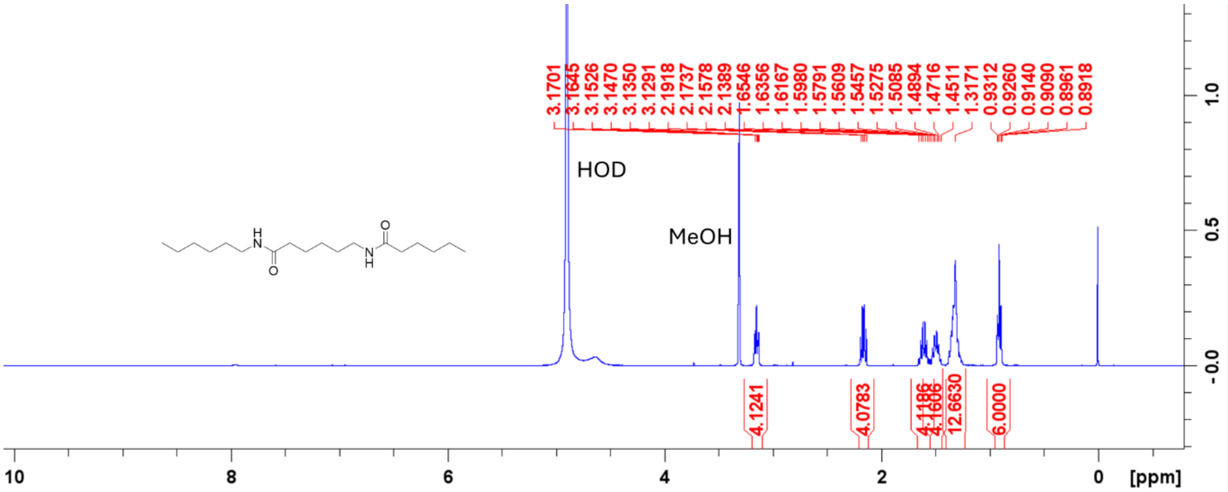

Figure S11. ^{1}H NMR spectrum (400 MHz, MeOD) of 1.

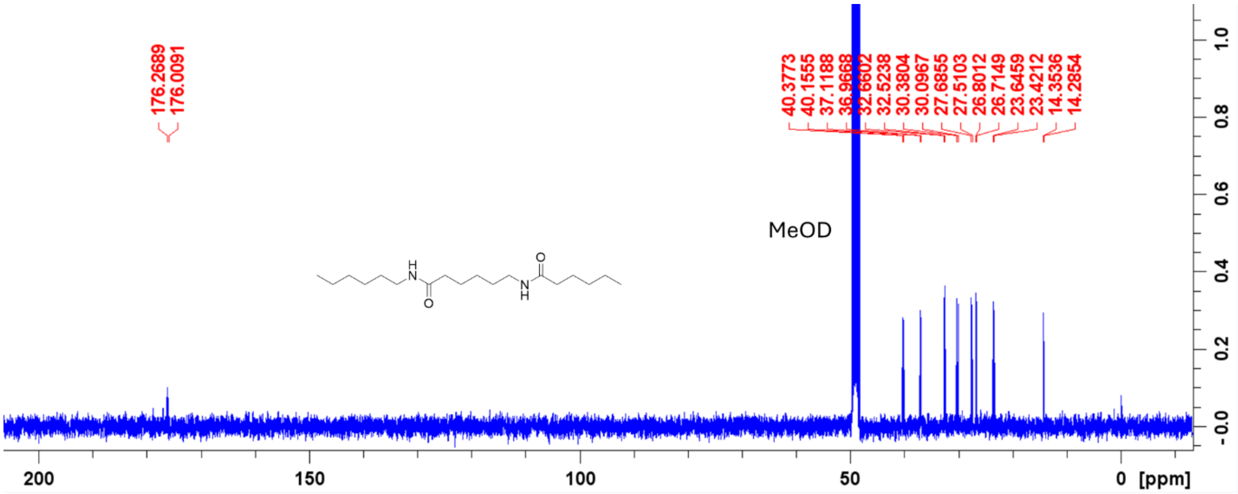

Figure S12. ^{13}C NMR spectrum (101 MHz, MeOD) of 1.

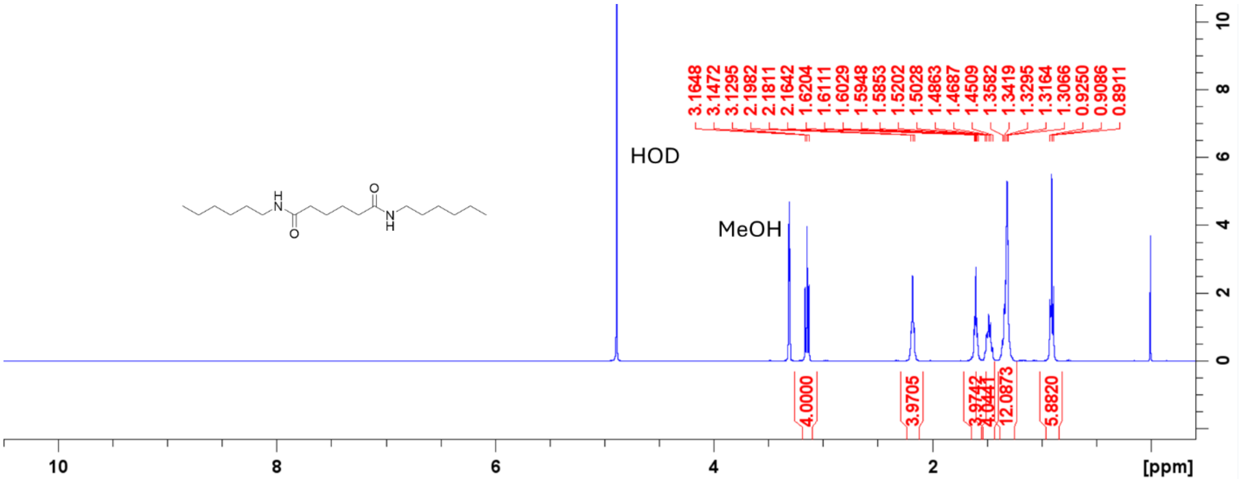

Figure S13. **^{1}H NMR spectrum (400 MHz, MeOD) of 2.**

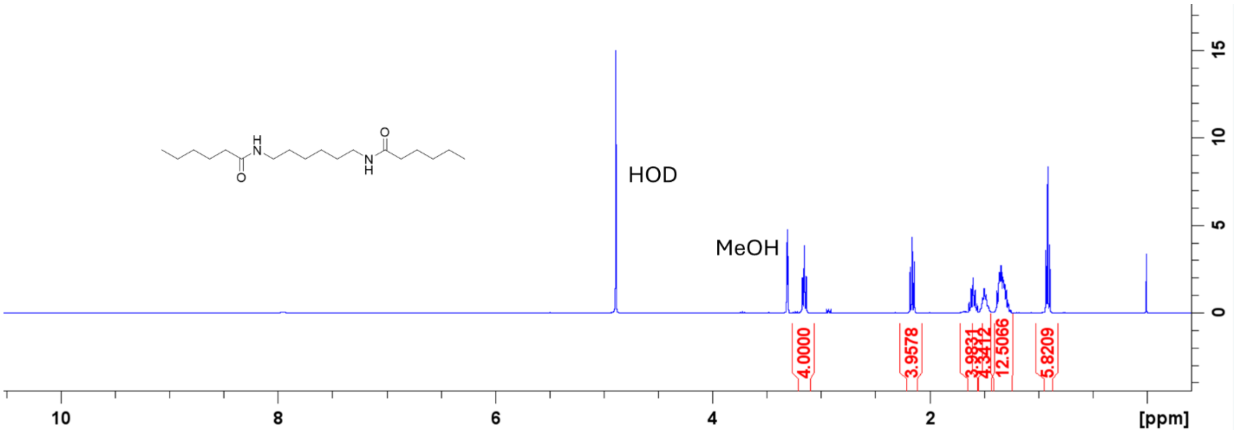

Figure S14. **^{1}H NMR spectrum (400 MHz, MeOD) of 3.**

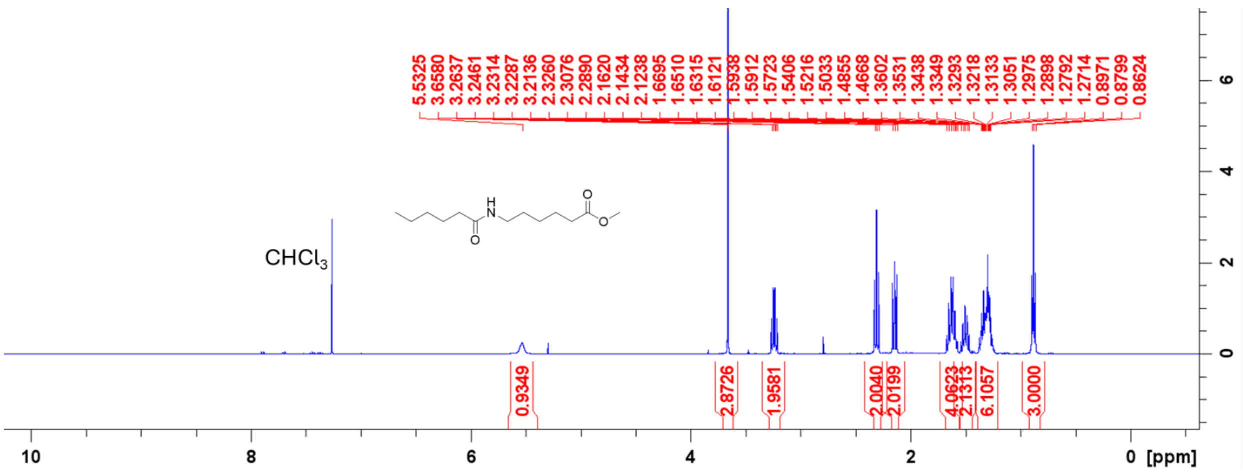

Figure S15. ¹H NMR spectrum (400 MHz, $CDCl_3$) of 5.

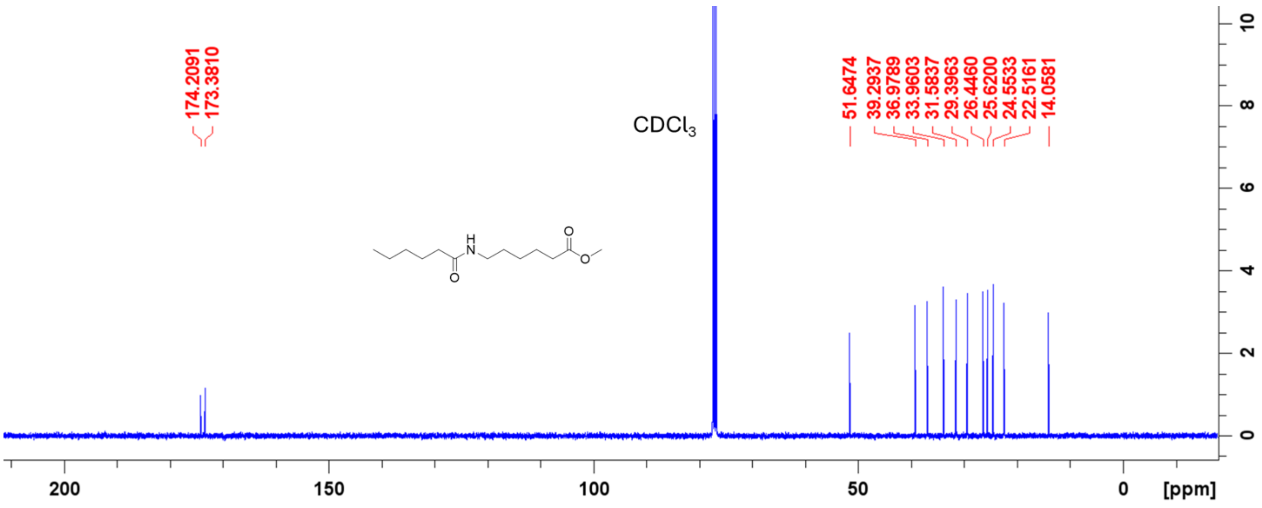

Figure S16. ¹³C NMR spectrum (101 MHz, $CDCl_3$) of 5.

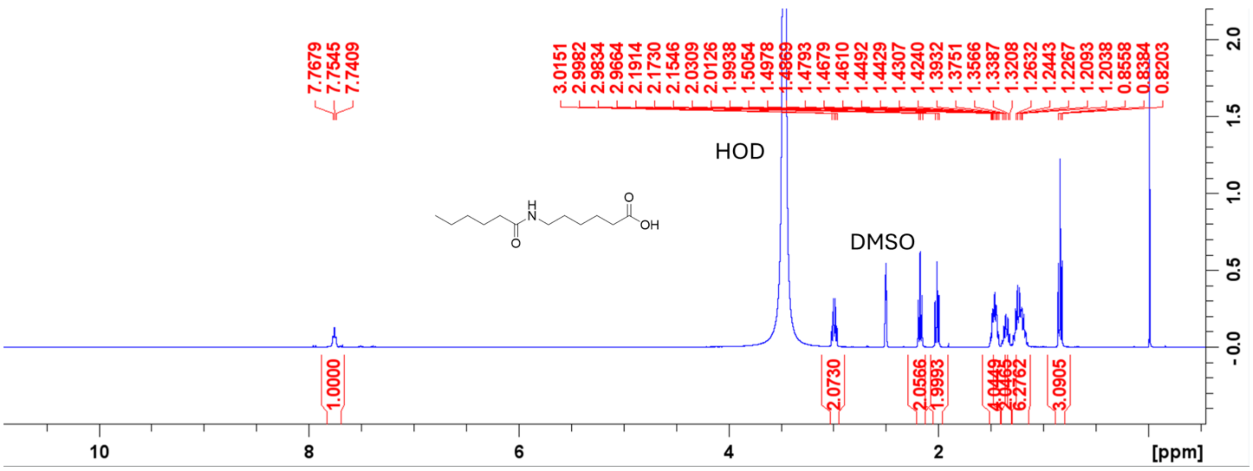

Figure S17. ^{1}H NMR spectrum (400 MHz, DMSO) of 1a.

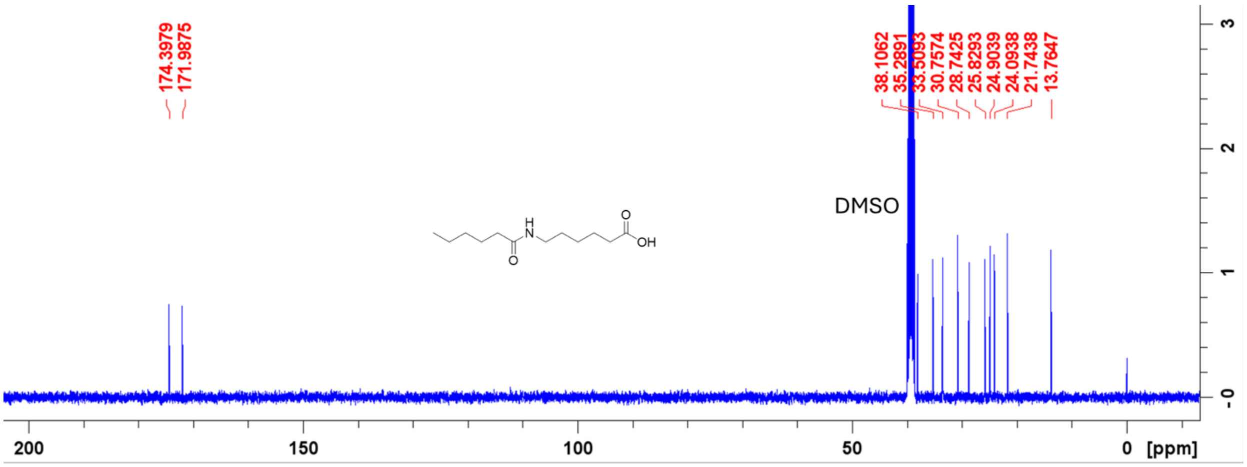

Figure S18. ^{13}C NMR spectrum (101 MHz, DMSO) of 1a.

References

[1] A. M. M. Rangaswamy, P. Navals, E. W. J. Gates, S. Shad, S. K. I. Watt, J. W. Keillor, “Structure–activity relationships of hydrophobic alkyl acrylamides as tissue transglutaminase inhibitors” *RSC Med. Chem.* **2022**, *13*, 413–428.

[2] M. M. Reynolds, Z. Zhou, B. K. Oh, M. E. Meyerhoff, “Bis-diazeniumdiolates of Dialkyldiamines: Enhanced Nitric Oxide Loading of Parent Diamines” *Org. Lett.* **2005**, *7*, 2813–2816.

[3] C. Gunanathan, Y. Ben-David, D. Milstein, “Direct Synthesis of Amides from Alcohols and Amines with Liberation of H2” *Science* **2007**, *317*, 790–792.

[4] E. L. Bell, G. Rosetto, M. A. Ingraham, K. J. Ramirez, C. Lincoln, R. W. Clarke, J. E. Gado, J. L. Lilly, K. H. Kucharzyk, E. Erickson, G. T. Beckham, “Natural diversity screening, assay development, and characterization of nylon-6 enzymatic depolymerization” *Nat. Commun.* **2024**, *15*, 1217.
